# Dynamic changes of acquired maternal SARS-CoV-2 IgG in infants

**DOI:** 10.1038/s41598-021-87535-x

**Published:** 2021-04-13

**Authors:** Xia Wang, Pu Yang, Junwen Zheng, Pin Liu, Cong Wei, Juanjuan Guo, Yuanzhen Zhang, Dongchi Zhao

**Affiliations:** 1 Department of Pediatrics, Children’s Digital Health and Data Center, Zhongnan Hospital of Wuhan University, Wuhan, 430071 Hubei China; 2 Department of Obstetrics and Gynecology, Zhongnan Hospital of Wuhan University, Clinical Medicine Research Center of Prenatal Diagnosis, Birth Health in Hubei Province, Wuhan, China

**Keywords:** Infectious diseases, Viral infection, Immunology, Microbiology

## Abstract

At present, there are still ambiguous reports about the perinatal infection of infants born to mothers infected with severe acute respiratory syndrome coronavirus 2 (SARS-CoV-2). The dynamic characteristics of infantile serum antibodies born to mother with SARS-CoV-2 has not been well described. In this study, we analyzed the seroconversion of 27 newborns born to 26 pregnant women infected with SARS-CoV-2. The SARS-CoV-2 IgG positive rate of parturient was 80.8%, and half of their infants obtained maternal IgG. IgG transfer rates were 18.8% and 81.8% in those infants whose mother infected less and more than 2 weeks before delivery. In the first two months of life, the IgG level of infants dropped sharply to one tenth of that at birth. These results suggest that maternal SARS-CoV-2 IgG provides limited protection for infants.

## Introduction

Pregnant women seem to be more vulnerable to coronavirus infected disease 2019 (COVID-19) caused by severe acute respiratory syndrome coronavirus 2 (SARS-CoV-2) infection due to the characteristic immune responses during pregnancy^1, 2^. It has been found that more than 13% admitted pregnant women were symptomatically with SARS-CoV-2 infection^3^. Vertical transmission of SARS-CoV-2 has not yet been detected, whereas perinatal transmission has been suspected in a few cases^4–9^. Due to the limitations of viral nucleic acid detection, SARS-CoV-2 serological antibody’s detection plays an important role in the diagnosis of pregnant women and their newborns^10^.

It is well known that when a woman was infected with a particular virus during pregnancy, the fetus can obtain specific maternal IgG through placental transport, which plays a passive immune role to protect the baby from virus infection after birth^11^. Changes of the level of baby’s specific antibodies can also prompt the intrauterine exposure risk. While the maternal gestational age (GA) at infected time and the duration of infection before delivery may affect their babies’ seroconversion. The dynamic changes of antibodies against SARS-CoV-2 were different from those of ordinary respiratory viruses^12–14^. The acquisition, maintenance time and protective effect of maternal antibody in infants are still unclear^13, 15–17^. Here, we described the serodynamic results of pregnant women with SARS-CoV-2 infection and their infants delivered in Zhongnan Hospital of Wuhan University in Wuhan, China.

## Results

### Objects and characteristics

Between January 27, 2020 and May 10, 2020, 26 pregnant women were confirmed to be infected with SARS-CoV-2 by laboratory evidences, and their 27 babies were tested for IgM and IgG antibodies against SARS-CoV-2 by chemiluminescence method. The positive rate of viral pneumonia-like in computed tomography (CT), SARS-CoV-2 seroconversion and viral nucleic acid test were 88.4% (23/26), 80.8% (21/26) and 34.6% (9/26) respectively in parturient women, while all infants were negative in SARS-CoV-2 nucleic acid test at birth. The age range of the mothers was 22–41 years, and the range of gestational age at admission was 31^6+^ to 41^1+^ weeks. There were five pregnant women with SARS-CoV-2 infection in the second trimester and 21 cases infected in the third pregnancy. 13 pregnant women had COVID-19-like symptoms such as fever and/or cough before delivery. Of the 27 infants, 21 were full-term, the other 6 were preterm including 1 pair of twins. The infants were normal at birth with Apgar scores all over 7. All infants were separated from their mothers immediately and were not breastfed before SARS-CoV-2 antibodies testing. The median time from onset of SARS-CoV-2 infection to delivery of parturient women was 10.5 days (1–107 days), and the primary seroconversion of IgG and IgM were 80.8% (21/26) and 53.9% (14/26) respectively (Table 1).

**Table 1:** Clinical data of mothers and infants recruited.

	Infants (n = 27)	Mothers (n = 26)	P value
**Sex**			
Male, n (%)	12 (44.4)	/	
Female, n (%)	15 (57.7)	/	
**Median GA, weeks (range)**	38.6 (31^+6^ ~ 41^+1^)		
< 37w, n (%)	6 (22.2)	/	
≥ 37w, n (%)	21 (80.8)	/	
**Median birth weight, g (range)**	2880 (1490 ~ 3850)	/	
< 2500 g, n (%)	7 (25.9)	/	
≥ 2500 g, n (%)	20 (74.1)	/	
Apgar score < 7 at 1.5 min, n (%)	0 (0), 0 (0)	/	
**Delivery mode**			
Eutocia, n (%)	4 (14.8)	4 (15.4)	
Cesarean, n (%)	23 (85.2)	22 (84.6)	
Median age, years (range)	/	31 (22 ~ 41)	
With symptom of COVID-19, n (%)	0 (0)	13 (50.0)	
CT scan with viral pneumonia, n (%)	0 (0)	23 (88.4)	
SARS-CoV-2 nucleic acid (+), n (%)	0 (0)	9 (34.6)	
**GAO**			
< 28w	/	5 (19.2)	
≥ 28w	/	21 (80.8)	
**Anti-SARS-CoV-2 IgG and IgM**			
IgM(−)/IgG (−),n (%)	15 (55.6)	5 (19.2)	0.0103
IgM (+)/IgG (−),n/N (%)	0 (0)	0 (0)	
IgM (−)/IgG ( +),n/N (%)	12 (44.4)	7 (26.9)	0.2492
IgM (+)/IgG (+),n/N (%)	0 (0)	14 (53.8)	< 0.0001
Total IgM ( +)	0 (0)	14 (53.8)	< 0.0001
Total IgG ( +)	12 (44.4)	21 (80.8)	0.0244
**IgG positive rate (%)**			
DMOD ≤ 14d, n/N (%)	3/16 (18.8)*	10/15 (66.7)	0.0113
DMOD > 14d, n/N (%)	9/11 (81.8)**	11/11 (100)	0.4762
GAO < 28w, n/N (%)	3/5 (60.0)	5/5 (100)	0.4444
GAO ≥ 28w, n/N (%)	9/22 (40.9)	16/21 (76.2)	0.0305

Between * and **, p value = 0.002.

*n/N* number of positive or negative cases/number of test cases, *CT* computed tomography, *DMOD* days from mother's onset to delivery, *GAO* gestational age at onset, *GA* gestational age.

### Detection and seroconversion of mothers and infants

Wuhan was the epicenter of SARS-CoV-2 outbreak, and from February to June in 2020, all pregnant women need to undergo the throat swab SARS-CoV-2 polymerase chain reaction with reverse transcription (RT-PCR) detection and lung CT examination before delivery. Since serological testing had not been widely used until March 2020, serum SARS-CoV-2 antibody detection was carried out in two stages: mothers who delivered before March 2020 received antibody testing in the follow-up stage after delivery, and pregnant women who gave birth after March 2020 were detected antibody before delivery. 11 pregnant women,who had positive of SARS-CoV-2 nucleic acid test or lung CT examination showed lung viral pneumonia-like changes during pregnancy, underwent serology testing 1–8 days before delivery. The median time from infection to delivery was 70 days (6–107 days), and 69 days (16–99 days) from infection to antibody detection day. All those mothers were IgG positive (11/11, 100%), 63.6% cases were IgM positive (7/11). Their 11 infants were tested for antibody 1–2 days after birth,and the median time from mother’s infection to infant’s first time of antibody detection was 71 days (7–108 days).The IgG was positive in 9 (9/11, 81.8%) infants born to this group mothers. 15 mothers confirmed with SARS-CoV-2 infection before delivery with symptoms, or without symptoms but with lung viral pneumonia-like changes, were tested for SARS-CoV-2 antibodies after delivery. The median time from infection to delivery was 4.5 days (1–15 days), and 64.5 days (36–81 days) from infection to antibody detection day. Among this group of mothers, 40% (6/15) were IgM positive, and 66.7% (10/15) were IgG positive. Their 16 infants of this group were tested 54–80 days after birth, and the median time from mother’s infection to infant’s first time of antibody detection was 69 days (57–83 days).Of these 16 infants, only 2 cases (12.5%, 2/16) were IgG positive.

Of 21 serum positive mothers, 53.8% (14 /21) cases were both IgG and IgM positive. There were 26.9% cases (7/21) with single IgG positive and there was no single IgM positive case. 12 infants (12/27, 44.4%) were IgG positive, and none of them was IgM positive. 5 mothers infected in the second trimester were all IgG positive, while their babies with 60% IgG positive rate. Among 21 mothers infected in the third trimester, 17 (81.0%) were IgG positive, and 9 infants (40.9%) were IgG positive (Table 1). Of the 22 infants born to 21 IgG-positive mothers, only 11 (50.0%) were IgG positive.

### Factors related to infantile acquisition of maternal IgG

**Figure 1:**
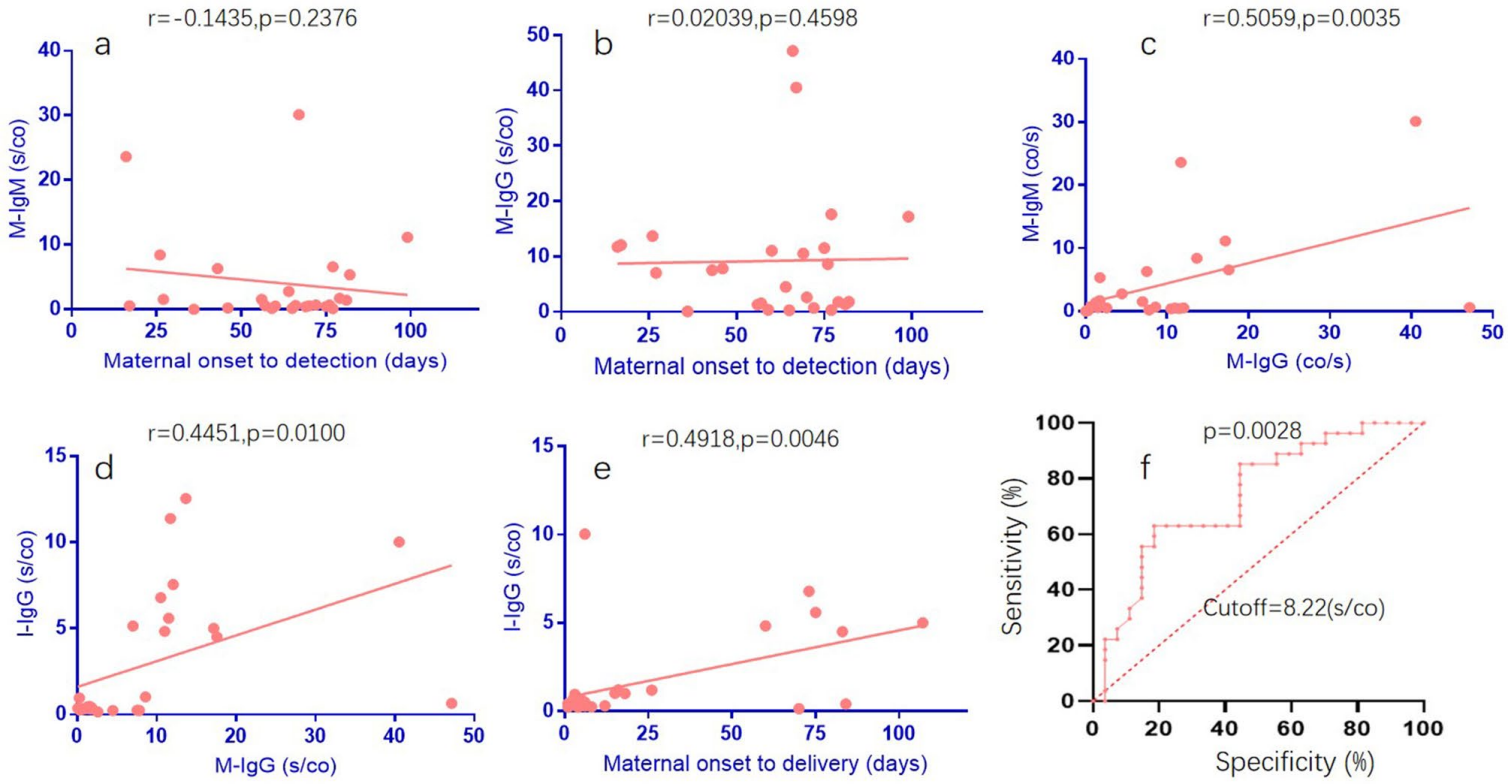
Dynamic characteristics of serum antibodies in pregnant women and infants. (**a**) Correlation between maternal infection time and serum IgM titer in pregnant women (M-IgM); (**b**) Correlation between maternal infection time and serum IgG titer in pregnant women (M-IgG); (**c**) correlation between the maternal serum IgG and IgM titer in pregnant women; (**d**) correlation between maternal serum IgG and infant IgG titer (I-IgG); (**e**) correlation between the maternal infection time before delivery and the IgG titer of infants; (**f**) predicting the sensitivity and specificity of maternal antibodies transferring into infants. Pearson test was used to analyze the correlation between the two groups, P < 0.05 was considered to be statistically significant.

To further understand the serodynamic changes of IgG and IgM in mothers and infants, we compared the correlation between maternal antibody level and infant serum conversion. As shown in Fig. 1a,b, there was no correlation between duration of maternal infection time and the titer of serum IgM and IgG (Fig. 1a,b). And the levels of IgG were positive correlated to that of IgM (p = 0.0035) (Fig. 1c). We also compared the correlation of the maternal serum antibodies titer with that of their infants, and found that the serum IgG titer of infants was positive correlated to that their mothers (p = 0.01) (Fig. 1d). A similar rule was found in the positive correlation between the serum antibody level of infants and the time of infection of mothers before delivery (Fig. 1e).

According to the time from onset of SARS-CoV-2 infection to delivery of mothers, we divided this group of data into 2 groups which were within 14-day and more than 14-day. The IgG seroconversion rates of mothers were 66.7% (10/15) and 100% (11/11), while the infants’ IgG positive rates were 18.8% (3/16) and 81.8% (9/11), respectively (p value = 0.002). Then we analyzed the relationship between the maternal and infantile IgG antibody titers and found that the two showed a positive correlation (Fig. 1d). The maternal IgG antibody titer was used to predict the positive of infantile IgG after birth (> 1 s/co), with a cutoff value of 8.22 s/co, which had a sensitivity of 84.3% and a specificity of 93.3% (Fig. 1f).

### Effects of mothers with and without symptoms on IgG conversion in infants (Table 2)

**Table 2:** Clinical characteristics of SARS-CoV-2 infected mothers with or without symptoms.

	With symptoms (13)	Asymptomatic (13)	P value
Mothers			
Median age, years (range)	31 (27 ~ 36)	30 (22 ~ 41)	0.4828
CT scan with viral pneumonia, n (%)	13 (100)	10 (76.9)	0.22
SARS-CoV-2 nucleic acid( +), n (%)	6 (46.2)	3 (23.1)	0.411
DMOD, days (range)	12 (1 ~ 107)	6 (3 ~ 84)	0.9492
Results of anti-SARS-CoV-2 Abs			
IgM(−)/IgG (−), n (%)	0 (0)	5 (38.5)	0.008
IgM ( +)/IgG (−), n/N (%)	0 (0)	0 (0)	
IgM (−)/IgG ( +), n/N (%)	3 (23.1)	4 (30.8)	
IgM (+)/IgG (+), n/N (%)	10 (76.9)	4 (30.8)	
Total IgM ( +)	10 (76.9)	4 (30.8)	0.0472
IgM titer (s/co)	4.79 ± 1.71	3.5 ± 2.30	0.6526
Total IgG ( +)	13 (100)	8 (61.5)	0.0391
IgG titer (s/co)	10.84 ± 3.07	7.39 ± 3.17	0.4414
IgG conversion rate of infants, n/N (%)	7/14 (50.0)	5/13 (38.5)	0.7036

*DMOD *days from mother's onset to delivery, *CT* computed tomography.

The seroconversion rate of mothers with symptoms before delivery was significantly higher than that of mothers without symptoms, and the IgM seroconversion rate of asymptomatic mothers was lower. However, there was no significant difference of IgG or IgM titer between these two groups of data.Whether mothers had symptoms did not affect the IgG conversion rate of their infants (50.0% in symptomatic group vs 38.5% in asymptomatic group).

### Serodynamic characteristics of maternal IgG

**Figure 2:**
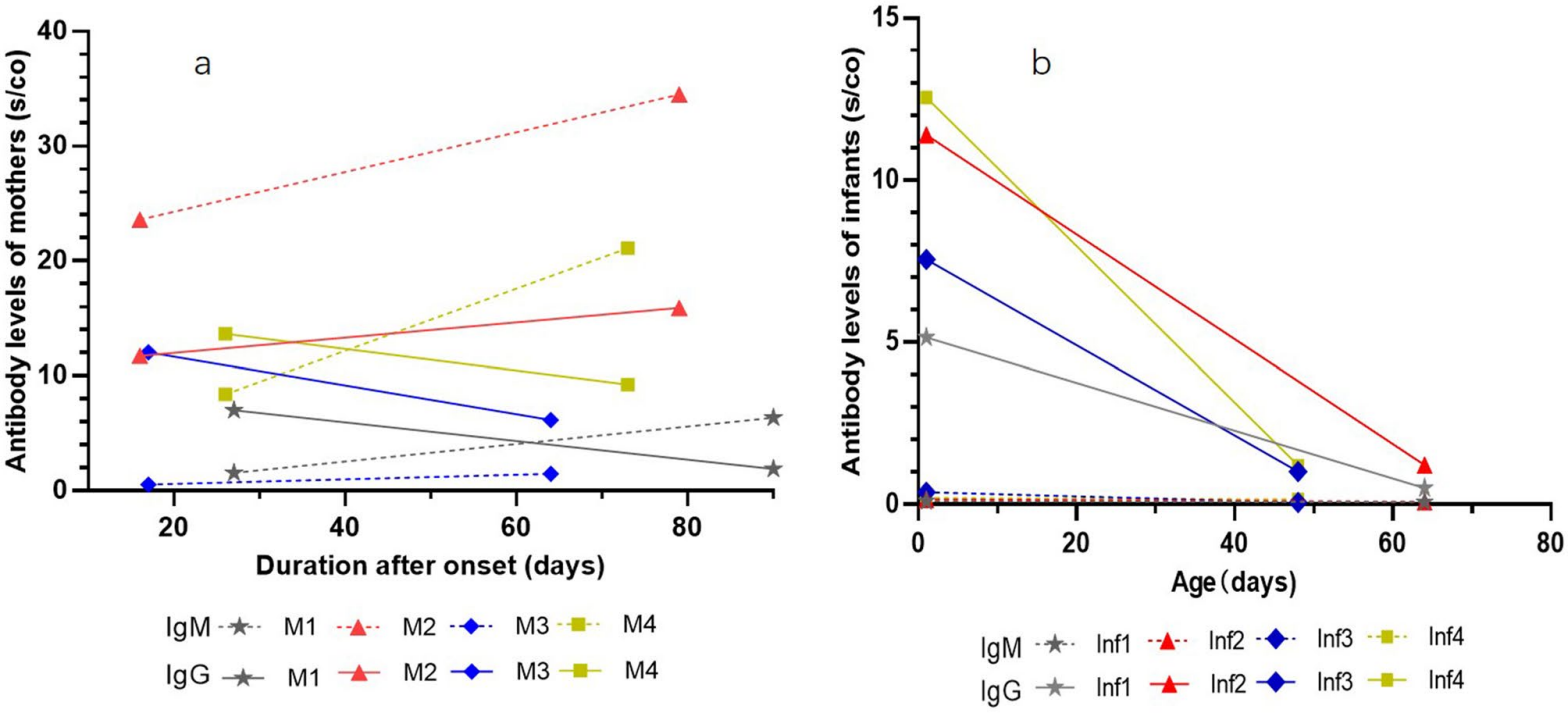
Dynamic changes of serum antibodies to SARS-CoV-2 in 4 COVID-19 mothers and their 4 infants. Antibody concentration ≥ 1.0 s/Co was defined as positive. Solid lines mean levels of IgG, dotted lines mean levels of IgM. *M* cases of mothers, *Inf* cases of infants. (**a**) antibodies levels of mothers; the serum antibodies detection interval of mother 1 was on the day of 27 and 91, mother 2 on the day of 16 and 71, mother 3 on the day of 17 and 64, and mother 4 on the day of 26 and 73 after onset. (**b**) antibodies levels of infants; the serum antibodies detection interval of four infants was on the day at birth and the day of 63, 63, 46 and 31 after birth, the same day with their mothers respectively. All 4 infants were formula fed.

In this study, four mothers were performed twice quantification tests of serum antibodies (Fig. 2a). They all had symptoms of fever or cough at the beginning of the onset with typical COVID-19 pneumonia findings of chest CT images. The median interval time between detection and onset of two tests were 26.5 days (17–46 days) and 72 days (62–91 days). The average serum titer of IgG was 11.1 s/co and 8.3 s/co, and the average titer of IgM was 8.5 s/co and 13.6 s/co respectively. The antibodies were also detected twice in their 4 infants on the first day and the day of 31–63 after birth (Fig. 2b). The IgM levels of all infants were below threshold, and the average IgG levels were 9.2 s/co and 1.0 s/co respectively. Infantile IgG levels decreased sharply in the first two months of life, accounting for only 10.7% of the titer at birth (Table 3).

**Table 3:** Detailed follow-up data of 4 mothers and 4 infants.

	Case1	Case2	Case3	Case4
Date of COVID-19 onset	20th Jan	31th Jan	15th Feb	8th Feb
GA at onset of COVID-19 (weeks)	36^+5^	37^+1^	35^+5^	28^+2^
Date of delivery	16th Feb	16th Feb	3rd Mar	5th Mar
GA at delivery (weeks)	37^+4^	39^+3^	38^+2^	32
Date of 1st Ab detection	16th Feb	16th Feb	3rd Mar	5th Mar
Delivery mode	Cesarean	Cesarean	Cesarean	Cesarean
Maternal 1st IgM (s/co)	1.56	23.6	0.56	8.4
Maternal 1st IgG (s/co)	7.01	11.74	12.06	13.67
Infantile 1st IgM (s/co)	0.1	0.15	0.38	0.2
Infantile 1st IgG (s/co)	5.14	11.39	7.55	12.55
Date of 2nd Ab detection	19th Apr	19th Apr	19th Apr	21th Apr
Days between 1st to 2nd Ab detection (days)	63	63	47	47
Maternal 2nd IgM (s/co)	6.38	34.51	1.48	21.11
Maternal 2nd IgG (s/co)	1.92	15.93	6.18	9.25
Infantile 2nd IgM (s/co)	0.07	0.08	0.06	0.15
Infantile 2nd IgG (s/co)	0.5	1.21	1.01	1.2
Infantile 1st IgG/Maternal 1st IgG (%)	73.32%	97.02%	62.60%	91.81%
Infantile 2nd IgG/Maternal 2nd IgG (%)	26.04%	7.60%	16.34%	12.97%

*GA* gestational age, *Ab* antibody.

## Discussion

The impacts of maternal SARS-CoV-2 infection on the fetus may include vertical transmission, abnormal intrauterine growth, abortion and stillbirth^9, 18–20^. At present, there are few studies focusing on serodynamic of infants born to mothers with SARS-CoV-2 infection during postpartum period. Due to the role of placental barrier, rare reports include neonatal test results for SARS-CoV-2 with positive cases so far^1^. Our data showed that no positive results of IgM were found in infants born to mothers with SARS-CoV-2, regardless of the mother's clinical symptoms, antibody titer and infection during the second or third trimester. And all infants were negative in SARS-CoV-2 nucleic acid test by RT-PCR detection of throat swabs at birth. Although the absence of IgM and a negative SARS-CoV-2 PCR at the time of birth cannot fully exclude an intra-uterine COVID-19 infection of the infants. We speculated that the SARS-CoV-2 IgG in infants can be used to observe whether they has acquired the passive immunity in this study.

In this study, we report antibody responses to SARS-CoV-2 in 26 pregnant women with COVID-19 and the seroconversion of their 27 babies. The probability of infantile acquisition of maternal antibody was related to the infection time before delivery and the serum antibody concentration of their mothers. The cutoff value was 8.22 s/co in mother, which had a sensitivity of 84.3% and a specificity of 93.3% for predicting postnatal infantile IgG positive (> 1 s/co). These results may be related to the dynamics of mother’s IgG. Many studies has found that SARS-CoV-2 -IgG peaked around weeks three to seven post-symptom onset then plateaued, generally persisting for at least eight weeks^21^. In the first two months of life, the IgG level of infants dropped sharply, only one tenth of that at birth. The IgM positive rate was 53.8% in mothers and negative in all infants. Although it is not yet clear whether this SARS-CoV-2-IgG has a neutralizing capacity, these results imply that maternal IgG provided limited protection for their infants.

There are many factors that affect infants to obtain maternal passive immunity. Despite the abundance of antibodies produced by prenatal infection of mothers, infants did not obtain the IgG titer in proportion to their mothers due to the short time period between maternal infection and delivery. This is consistent with our expectations, in which the longer the mother infected the virus before delivery, the higher the seroconversion rate of the maternal IgG, and the greater the probability of infants obtaining maternal antibody.

Mothers who had been infected for more than two weeks give their babies more adequate antibody titers. However, the longer the intrauterine exposure, the more likely to affect the development of fetus, which involve other high-risk factors^21^. The maternal IgG transfer efficiency was also depended on the mother's immune response to SARS-CoV-2. The IgG titer of infants born to high titer IgG mothers also increased. Although the neutralizing capacity of this IgG is not yet clear, the level of SARS-CoV-2 neutralizing antibodies was found to correlate with a wide range of specific antibodies^21, 22^. Most studies suggested that neutralization ability correlated positively with total virus-specific IgG^22–24^. Thus, we speculate that the duration of effective concentration of maternal SARS-CoV-2 IgG in infants may be related to their risk of infection after birth. Vertically transferred immunity can dominantly influence the response of offspring to vaccination. High-titer maternal antibodies have often been associated with diminished primary antibody response of infants to vaccines, although the T cell response is usually unaffected. Interference of infant serological response is observed for live and inactivated vaccines, although the reduction magnitude is variable between studies and individual vaccines^25–29^. It is generally believed that maternal antibodies disappear gradually in 12–18 months after birth. However, the maternal protective effect of against SARS-CoV-2 in infants was rapidly eliminated naturally after birth. These results probably help us understand the risk of infantile vulnerability and is hoped to be helpful to children's SARS-CoV-2 vaccination procedures in the future.

In summary, the infection time and antibody titer of mothers before delivery affected the acquisition maternal IgG in their infants, and the passive immunity lasted for a short time and disappeared in 2 months after birth. Our study has some limitations. This study is a retrospective study with a small sample size. Since the serological test had not be widely used at the early stage of the epidemic, no comparison was made between the antibody titer of the mother before delivery and the antibody titer of the baby at birth. The IgG neutralizing capacity were not studied, whether the specific antibody in the baby can play a protective effect needs further observation, and more data are needed for following research.

## Methods

### Objective

In this paper, the serum antibodies of 26 pregnant women who were diagnosed with SARS-CoV-2 infection and 27 infants (1 pair of twins) in Zhongnan Hospital of Wuhan University from January 27, 2020 to May 10, 2020 were analyzed. The infants were admitted to neonatology department for serological antibody test after birth. The mother began breastfeeding if they were asymptomatic after 2 weeks of isolation and negative result of viral nucleic acid detection. During the follow-up with 3 months after the birth of the infant, the results of serum antibody test of SARS-CoV-2 in 4 mothers and 4 infants were obtained.

### The timing of serology testing for mothers and infants

Since the serological test had not be widely used until March 2020, the primary time of SARS-CoV-2 antibody detection was performed as two groups. 11 pregnant women underwent serological testing 1–8 days before delivery. The median time from infection to delivery was 70 days (6–107 days), and the median time from infection to antibody detection was 69 days (16–99 days).The 11 infants were tested for antibody 1–2 days after birth, and the median time from mother’s infection to infant’s initial antibody detection was 71 days (7–108 days) 0.15 mothers who were confirmed with SARS-CoV-2 infection before delivery underwent antibodies test after delivery. The median time from infection to delivery was 4.5 days (1–15 days), and the median time from infection to antibody detection was 64.5 days (36–81 days). The 16 infants of this group mothers were tested 54–80 days after birth,and the median time from mother’s infection to infant’s initial antibody detection was 69 days (57–83 days).

### Detection method of serum IgM and IgG antibodies

The serum IgM and IgG levels were quantified by Axceed 400t automatic chemiluminescent immunoanalyzer (Tianjin boassi Biotechnology Co., Ltd. China) and matching reagents.The test was carried out according to the operating instructions of the kit (chemiluminescence method). The test result ≥ 1.0 s/co was defined as positive.

### Detection of SARS-CoV-2 nucleic acid

The total RNA was extracted from the pharyngeal swabs and the SARS-COV-2 nucleic acid was detected by qRT-PCR automatically. Operation was under the instruction of China's novel coronavirus (ORF1ab/N) nucleic acid detection kit (Shanghai BioGerm Medical Technology Co., Ltd. China), based on the guidelines of the Center for Disease Control and Prevention in China. The same test system was used throughout the study.

### Definition of SARS-CoV-2 infection in mothers

Either positive results of nucleic acid or specific antibodies to SARS-CoV-2 detection.

### Statistics

Statistical analyses were performed using a two-tailed unpaired t-test, Fisher’s test. P value < 0.05 was considered significant. All statistical calculations were performed with Prism 8 (GraphPad Software Inc., La Jolla, CA).

### Ethical approval and informed consent

This study was registered as a clinical study with the Chinese Clinical Trial Registry (ChiCTR-ORC-16008872), and the Medical Ethical Committee of Zhongnan Hospital of Wuhan University approved the study (approval no. 2015019). Informed consent was signed and obtained from all subjects (pregnant women and guardians). The protocol and procedures employed were followed the principles outlined in the Helsinki Declaration.

### Ethics statement

The protocol and procedures employed were reviewed and approved by the appropriate institutional review committee and followed the principles outlined in the Helsinki Declaration.

## References

[1] Wastnedge EAN (2021). Pregnancy and COVID-19. Physiol. Rev..

[2] Ellington S (2020). Characteristics of women of reproductive age with laboratory-confirmed SARS-CoV-2 infection by pregnancy status—United States, January 22-June 7, 2020. MMWR Morb. Mortal Wkly. Rep..

[3] Sutton D, Fuchs K, D'Alton M, Goffman D (2020). Universal screening for SARS-CoV-2 in women admitted for delivery. N. Engl. J. Med..

[4] Zeng H (2020). Antibodies in infants born to mothers with COVID-19 pneumonia. JAMA.

[5] Yu N (2020). Clinical features and obstetric and neonatal outcomes of pregnant patients with COVID-19 in Wuhan, China: A retrospective, single-centre, descriptive study. Lancet Infect. Dis..

[6] Lamouroux A, Attie-Bitach T, Martinovic J, Leruez-Ville M, Ville Y (2020). Evidence for and against vertical transmission for severe acute respiratory syndrome coronavirus 2. Am. J. Obstet. Gynecol..

[7] Dong L (2020). Possible vertical transmission of SARS-CoV-2 from an infected mother to her newborn. JAMA.

[8] Yang P (2020). Clinical characteristics and risk assessment of newborns born to mothers with COVID-19. J. Clin. Virol..

[9] Chen H (2020). Clinical characteristics and intrauterine vertical transmission potential of COVID-19 infection in nine pregnant women: A retrospective review of medical records. Lancet.

[10] Xu Y (2020). Characteristics of pediatric SARS-CoV-2 infection and potential evidence for persistent fecal viral shedding. Nat. Med..

[11] Carsetti R (2020). The immune system of children: The key to understanding SARS-CoV-2 susceptibility?. Lancet Child Adolescent Health.

[12] Ozcurumez MK (2020). SARS-CoV-2 antibody testing—questions to be asked. J. Allergy Clin. Immunol..

[13] Long QX (2020). Antibody responses to SARS-CoV-2 in patients with COVID-19. Nat. Med..

[14] Weinstein MC, Freedberg KA, Hyle EP, Paltiel AD (2020). Waiting for certainty on Covid-19 antibody tests—At what cost?. N. Engl. J. Med..

[15] Liu P (2020). The immunologic status of newborns born to SARS-CoV2-infected mothers in Wuhan China. J. Allergy Clin. Immun..

[16] Kimberlin DW, Stagno S (2020). Can SARS-CoV-2 infection be acquired in utero?: More definitive evidence is needed. JAMA.

[17] Yu N (2020). No SARS-CoV-2 detected in amniotic fluid in mid-pregnancy. Lancet Infect. Dis..

[18] Baud D (2020). Second-trimester miscarriage in a pregnant woman with SARS-CoV-2 infection. JAMA.

[19] Khalil A (2020). Change in the incidence of stillbirth and preterm delivery during the COVID-19 pandemic. JAMA.

[20] Sentilhes L (2020). Coronavirus disease 2019 in pregnancy was associated with maternal morbidity and preterm birth. Am. J. Obstet. Gynecol..

[21] Post N (2020). Antibody response to SARS-CoV-2 infection in humans: A systematic review. PLoS ONE.

[22] Robbiani DF (2020). Convergent antibody responses to SARS-CoV-2 in convalescent individuals. Nature.

[23] Ni L (2020). Detection of SARS-CoV-2-specific humoral and cellular immunity in COVID-19 convalescent individuals. Immunity.

[24] Varnaite R (2020). Expansion of SARS-CoV-2-specific antibody-secreting cells and generation of neutralizing antibodies in hospitalized COVID-19 patients. J. Immunol..

[25] Kollmann TR, Marchant A, Way SS (2020). Vaccination strategies to enhance immunity in neonates. Science.

[26] Voysey M (2017). The influence of maternally derived antibody and infant age at vaccination on infant vaccine responses: An individual participant meta-analysis. JAMA Pediatr..

[27] Kim D, Huey D, Oglesbee M, Niewiesk S (2011). Insights into the regulatory mechanism controlling the inhibition of vaccine-induced seroconversion by maternal antibodies. Blood.

[28] Niewiesk S (2014). Maternal antibodies: Clinical significance, mechanism of interference with immune responses, and possible vaccination strategies. Front Immunol..

[29] Gostin LO, Salmon DA (2020). The dual epidemics of COVID-19 and influenza: Vaccine acceptance, coverage, and mandates. JAMA.

